# Surgical Antibiotic Prophylaxis Use and Surgical Site Infection Pattern in Dessie Referral Hospital, Dessie, Northeast of Ethiopia

**DOI:** 10.1155/2020/1695683

**Published:** 2020-03-18

**Authors:** Desye Misganaw, Bedilu Linger, Atinkut Abesha

**Affiliations:** ^1^Pharmacology and Toxicology Unit, Department of Pharmacy, College of Medicine and Health Science, Wollo University, Ethiopia; ^2^Department of Clinical Pharmacy, College of Medicine and Health Science, Wollo University, Ethiopia

## Abstract

**Background:**

Surgical site infections are the third (14%-16%) most frequent cause of nosocomial infections among hospitalized patients. They still form a large health problem and result in increased antibiotic usage, increased associated costs, and prolonged hospitalization and contribute to increased patient morbidity and mortality. Therefore, studies on surgical site infections and surgical antibiotic prophylaxis contribute to identifying surgical site infection rate and risk factor associated with it as well as for identifying the gap in surgical antibiotic prophylaxis practice.

**Objective:**

To assess surgical antibiotic prophylaxis practice and surgical site infection among surgical patients.

**Method:**

A hospital-based prospective observational study was conducted in 68 patients who underwent major surgery in Dessie Referral Hospital adult surgical wards between March 24 and April 25/2017. Descriptive and logistic regression analyses were performed to determine infection rate and risk factors for surgical site infections.

**Result:**

Assessment of 68 patients who underwent major surgery revealed an overall surgical site infection rate of 23.4%. Prophylactic antibiotics were administered for 59 operations; of these, 33 (48.6%) had inappropriate timing of administration. A combination of ceftriaxone and metronidazole 28 (47.46%) was frequently used. Factors associated with surgical site infection were wound class, patient comorbid condition, duration of the procedure, the timing of administration, and omitting prophylaxis use.

**Conclusion:**

This study indicated a higher rate of surgical site infection and also revealed that wound class, preexisting medical condition, prolonged duration of surgery, omitting of prophylaxis use, and inappropriate timing of administration were highly associated with surgical site infection.

## 1. Background

Surgical site infections (SSIs) are the third (14%-16%) most frequent cause of nosocomial infections among hospitalized patients and the primary (40%) cause of nosocomial infection in surgical patients [1]. SSIs have been categorized by the Center for Disease Control and Prevention (CDC) as either incisional (e.g., cellulitis of the incision site) or involving an organ or space (e.g., with meningitis). Incisional SSIs may be superficial (skin or subcutaneous tissue) or deep (fascial and muscle layers). Both types, by definition, occur by postoperative day 30. This period extends to 1 year in the case of deep infection, associated with prosthesis implantation [2]. Besides, the National Research Council, USA, developed a system for categorizing incisions based on the degree of contamination as clean, clean-contaminated, contaminated, and dirty wounds [3].

Globally, SSI has a 2.5% to 41.9% prevalence rate, and it is higher in developing countries like Ethiopia and Tanzania [4–8], inducing a substantial burden in terms of health cost and postoperative morbidity and mortality.

The development of postoperative site infection is related to the degree of bacterial contamination during surgery, the virulence of the infecting organism, and host defenses. Besides, risk factors for postoperative site infection can be classified according to operative and environmental factors and patient characteristics [9, 10].

Surgical antibiotic prophylaxis (SAP) is the use of antibiotics to prevent infections at the surgical site. SAP is an effective management strategy for reducing postoperative infections provided that appropriate antibiotics are given at the correct time for appropriate durations and appropriate surgical procedures [11]. However, in actual practice, there is considerable evidence that antibiotics are used excessively and inappropriately for the prevention of SSI [12]. These inappropriate surgical antibiotic prophylaxes such as inappropriate selection, timing, and duration are associated with an increase in the prevalence of antibiotic resistance, cause adverse drug reaction and increased risk of surgical site infections, i.e., fuel an ever-increasing need for new drugs, and contribute to the rising cost of medical care [13].

Studies on SSI and SAP contribute for identifying surgical site infection rate and risk factor associated with it as well as for identifying the gap in surgical antibiotic prophylaxis practice. Therefore, this study attempts to assess surgical antibiotic prophylaxis practice and prevalence of surgical site infection among surgical patients in Dessie Referral Hospital (DRH), which will be helpful to promote SSI control and rational antibiotic prophylaxis utilization.

## 2. Methods

### 2.1. Study Design and Area

A prospective cross-sectional study design was conducted in DRH Northeast Ethiopia from March 24 to April 25/2017. DRH is 401 km far from the capital city of Ethiopia, Addis Ababa. It is one of the biggest referral hospitals in Northeast Ethiopia and has different specialized service in five major departments: the Pediatric, Surgery, Gynecology, OPD, and Internal Medicine. It provides services for the patient living in Dessie town and referred from different parts of the region and provides local emergency services.

### 2.2. Sampling

The study was done on patients who underwent major surgery in the adult surgical ward of DRH during the study period. Those patients who received antimicrobials for therapeutic purposes preoperatively and enrolled patients who had shown signs and symptoms of infection within the first 48 hrs of admission were excluded from the study. A convenience sampling technique was used for those patients who fulfill the inclusion criteria.

The dependent variable in this study is SSI (presence/absence), and the independent variables are sociodemographic characteristics of the patients (age and sex), medical illness (DM, HTN, CVD, and others), preoperative hospital stay, duration of surgery, class of contamination of surgical site, type of operative procedure, antibiotic use (choice, dose, duration, frequency, and timing of administration), and postoperative hospital stay.

Timing of antibiotic administration was classified as follows: early prophylaxis (2-24 hours before surgery), preoperative prophylaxis (0-2 hours before surgery), perioperative prophylaxis (up to 3 hours after the first incision), and postoperative prophylaxis (greater than 3 hours after the first incision) [14].

### 2.3. Data Collection Procedure

Data collection format containing the variables to be measured was developed and used for the collection of data on sociodemographic characteristics, surgery-related parameters (preoperative, intraoperative, and postoperative data), and potential risk factors. Wound classification and diagnosis of SSI were done by a surgeon, gynecologists, and the attending physician.

Information about SSI was obtained through a medical chart review. Each patient was assessed from the time of admission until the time of discharge. Details that were recorded include the type of surgery, wound class, type and duration of operation, antimicrobial prophylaxis, preoperative hospital stay, and total hospital stay. The data quality was controlled before collection through pretesting and during and after collection through direct observation.

### 2.4. Limitation and Strength of the Study

Strength of the study: the study design is a prospective cross-sectional study, and primary data was obtained.

Limitation of the study: the study period was short, and the number of patients recruited was somewhat low.

### 2.5. Data Processing and Analysis

The collected data were filtered and categorized, and the results were analyzed using SPSS version 20.0 and interpreted and presented using tables and charts. Descriptive statistics (frequency, mean, and SD) were analyzed to determine the prevalence of the dependent variable. Two-way tables were used to summarize the data, and associations between categorical variables were determined using chi-square. A probability value of <0.05 was considered statistically significant. Furthermore, logistic regression analysis was used to determine relationships between a risk factor and SSI.

## 3. Results

A total of 68 patients were assessed for surgical site infection and surgical prophylaxis use as depicted in Table 1. 51.5% of them were males, and the rest were females within the age of 20-80. Regarding patient operational characteristics, as revealed in Table 2, the patients were hospitalized with a mean ± SD length of 2.98 ± 2.76 and 5.62 ± 2.99 days preoperatively and postoperatively, respectively. From the total patients, 12 (17.65%) were with the comorbid condition, and 16 (23.5%) had developed SSI. Moreover, 38 (55.9%) were emergency operations, and gastrointestinal (GI) was the most frequent, 21 (30.9%), surgical procedure. Of the total procedures, 25 (36.8%) were clean, and the rest (63.2%) accounts for clean-contaminated, contaminated, and dirty wounds as depicted in Table 2.

Antibiotic prophylaxis was administered in 59 (86.8%) operations and most, 26 (38.2%), of the drugs were given preoperatively, of which 55 (93.2%) were given in IV route as shown in Table 3. The mean ± SD duration of antibiotic prophylaxis was 3.89 ± 2.81 days (range 1-9 days). Besides, prophylaxis was extended over one day in 43 (63.2%) patients.

Among all patients given antibiotic prophylaxis, 18 (26.5%) patients used single prophylactic agents, and the rest took a combination of drugs as shown in Table 4. The most frequently administered prophylactic antibiotics were a combination of ceftriaxone and metronidazole 28 (47.46%).

**Figure 1:**
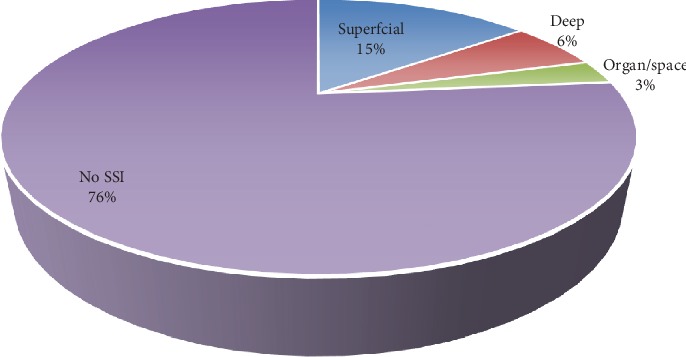
Surgical site infection rate.

Concerning the surgical site infection rate, 16 patients had developed infection, and the incidence rate was 23.5% as revealed in Figure 1. Of this, superficial, deep, and organ/space SSIs account 62.5%, 25.0% and 12.5%, respectively.

Different variables were analyzed to pinpoint the possible risk factors for surgical site infection as depicted in Table 5. Thus, a descriptive analysis of patient characteristics revealed that patients with older age (>68 yrs) had the highest percentage (50%) of infection among all age groups. Moreover, females (27.3%) had a higher percentage of infection than males (20%). Patients with the comorbid condition (*p* value, 0.00) and patients without prophylaxis (*p* value, 0.00) had a higher infection rate than patients with nil comorbid condition and who had prophylaxis, respectively. Also, patients who had a longer duration of the procedure (28%), dirty wound (50%), and postantibiotic administration of prophylaxis (27.6%) had a higher frequency of SSI compared to their category.

According to the result of binary logistic regression analysis, the following variables were statistically significant at (*p* < 0.05) level: patient comorbid condition, duration of procedure, wound class, prophylaxis use, and timing of administration, However, age, sex, nature of operation, preoperative hospital stay, and postoperative hospital stay duration were not significantly associated in this study (Table 6).

## 4. Discussion

SSIs are known to be one of the most common causes of nosocomial infections worldwide. They still form a large health problem and result in increased antibiotic usage, increased associated costs, prolonged hospitalization, and contribute to increased patient morbidity and mortality [15]. The rate of SSI varies greatly, from 2.5% to 41.9% as per different studies, worldwide and from hospital to hospital [16].

The rate of surgical site infections found in this study was 23.5%, which was higher than the finding of a similar study conducted in Tikur Anbessa Specialized Hospital [17] where the rate of infection reported was 17.9%. Moreover, it was still higher than the other studies carried out elsewhere in other countries such as India [16], Muhimbili [18], and Brazil where the infection rate of 16%, 20%, and 15% was reported, respectively. The higher rate of infection in this study could be explained by a lack of adequate infection control system, poor practices, and indiscriminate use of antibiotics.

The study also showed that prolonged duration of operation was a significant risk factor for SSIs. Thus, surgical procedures lasting greater than one hour had about 2 times more risk than procedures within an hour. This finding was comparable with a similar study done in Thailand (RR = 3.26, 95%CI = 1.44 − 7.52) and also supported by Peruvian hospital study [19]. Moreover, wound class was also found to be an important risk factor in the development of SSI. Hence, dirty had the highest odds (7.33) of becoming infected followed by the contaminated wound and clean-contaminated. This high rate of infection among former wound types would be because of the profound influence of endogenous contamination during the time of operation, and it is supported by other studies [20].

Besides, the study signified that comorbid conditions were an important risk factor and statically significant (OR = 5, *p* = 0.00) for the development of SSIs. This finding is also supported by different kinds of literature, which indicate that the prevalence of SSI is higher with HIV/AIDS and other immunosuppression-related conditions such as malignancy and diabetes mellitus [21, 22]. Besides, the study also revealed that the timing of antibiotic administration and the presence of antibiotic prophylaxis are critical in preventing surgical wound infections. Therefore, the administration of antibiotics postoperatively had 9.94 times the risk of SSI than the preoperative administration. Similarly, 13.24% of patients were not receiving surgical antibiotic prophylaxis and had 2.00 times the risk of SSI as compared with patients with antibiotic prophylaxis.

The use of antibiotics in surgical patients for the prophylaxis is a justifiable practice; however, appropriate route of administration, timing, and duration of prophylactic antibiotics should be chosen to achieve high plasma and tissue levels of antibiotics during and shortly after the surgical procedure when bacterial contamination is maximal [23, 24]. In this study, the majority of the antibiotics was administered IV (93.2%), which is consistent with evidence [23], and the most frequently administered antibiotics were ceftriaxone and metronidazole (47.1%) despite first-generation cephalosporins (like cefazolin) are recommended for SSIs [23, 25]. Still, different studies showed that first-generation cephalosporins (e.g., cefazolin) were the most commonly used drugs [26, 27]. The frequent use of the combination of ceftriaxone and metronidazole in this study is explained partly by the lack of availability of first-generation cephalosporins in the hospital.

SSI rate increases with age (higher incidence with patients above 68 years of age) and in patients who underwent emergency surgery, despite it were not statistically significant. However, no relationship was observed between the development of SSI and the gender of the patient (*p* = 0.808), which is in concordance with other studies [14]. These findings are also similar to other studies as increasing age is usually associated with a greater likelihood of certain chronic conditions and decreased immunity with delayed healing [22]. Besides, this increased rate of SSI during emergency surgery could be due to improper preparation and planning before the surgical procedure [14]. Therefore, antibiotic prophylaxis with proper timing has paramount importance in decreasing the incidence of SSI. In addition, considering the type of wound, duration of surgery and comorbid condition would also have vital role in minimizing SSI rate.

## 5. Conclusion

This study indicated a higher rate of surgical site infection. Wound class, preexisting medical condition, prolonged duration of surgery, absence of antibiotic prophylaxis, and early and delayed administration of antibiotics were statistically significant with surgical site infection. This study also indicated that the majority of the antibiotics were administered IV, and the most frequently administered were ceftriaxone and metronidazole.

## Figures and Tables

**Table 1:** Patient demographic characteristics.

Variables		Frequency (%)
Age	14-22	6 (8.82)
	23-31	26 (38.23)
	32-40	12 (17.65)
	41-49	4 (5.88)
	50-58	10 (14.70)
	59-67	6 (8.82)
	68-76	2 (2.94)
	77-85	2 (2.94)

Sex	Male	35 (51.47)
	Female	33 (48.53)

**Table 2:** Patient operation characteristics.

Variables		Frequency (%)
Patient comorbidity	Present	12 (17.65)
	Absent	56 (82.35)

Nature of operation	Elective	30 (44.1)
	Emergency	38 (55.9)

Surgical procedure	GI surgery	21 (30.9)
	Urologic surgery	11 (16.2)
	Neck surgery	11 (16.2)
	Gynecologic surgery	10 (14.7)
	Orthopedic surgery	7 (10.3)
	Other surgery	8 (11.8)

Wound class	Clean	25 (36.8)
	Clean-contaminated	22 (32.4)
	Contaminated	17 (25)
	Dirty	4 (5.9)

Duration of procedure	≤1 hr	43 (63.2)
	>1 hr	25 (36.8)

SSI	Yes	16 (23.5)
	No	52 (76.5)

Preoperative duration of hospitalization	≤1 day	30 (44.1)
	>1 day	38 (55.9)

Postoperative duration of hospitalization	≤3 days	37 (54.4)
	>3 days	31 (44.6)

**Table 3:** Characteristics of antibiotic prophylaxis administration.

Variables		Frequency (%)
Prophylaxis	Yes	59 (86.8)
	No	9 (13.2)

Timing of prophylaxis administration	Early	7 (10.3)
	Preoperative	26 (38.2)
	Perioperative	5 (7.4)
	Postoperative	21 (30.9)

Duration of SAP administration	≤1 day	16 (23.5)
	>1 day	43 (63.2)

Route	IV	55 (93.2)
	IV and PO	4 (6.8)

**Table 4:** Prophylactic drugs used in surgical patients.

Prophylaxis drug	Frequency (%)
Ceftriaxone	10 (14.7)
Cloxacillin	8 (11.8)
Ceftriaxone+metronidazole	28 (47.46)
Ceftriaxone+ampicillin	5 (7.4)
Ceftriaxone+gentamicin+metronidazole	4 (5.9)
Ceftriaxone+metronidazole+amoxicillin	4 (5.9)

**Table 5:** Risk factors and surgical site infection.

Risk factors		SSI		*χ* ^2^, *p* value•
		Yes (%)	No (%)	
Age (yrs)	14-22	1 (16.7)	5	27.58, 0.327
	23-31	7 (25)	21	
	32-40	2 (16.7)	10	
	41-49	1 (25)	3	
	50-58	2 (20)	8	
	59-67	1 (16.66)	5	
	68-76	1 (50)	1	
	77-85	1 (50)	1	

Sex	Male	7 (20)	28	0.59, 0.808
	Female	9 (27.3)	24	

Preoperative hospital stay	≤1	5 (16.7)	25	0.941, 0.332
	>1	11 (28.9)	27	

Nature of operation	Elective	6 (20)	24	0.941, 0.332
	Emergency	10 (26.3)	28	

Surgical procedure	GI surgery	6 (28.6)	15	11.059, 0.05
	Urologic	4 (36.4)	7	
	Neck	0 (0)	11	
	Gynecologic	3 (30)	7	
	Orthopedic	2 (28.6)	5	
	Others (BPH, amputation…)	1 (12.5)	7	

Duration of procedure	≤1 hr	9 (20.9)	34	4.765, 0.029
	>1 hr	7 (28)	18	

Wound classification	Clean	3 (12)	22	15.176, 0.002
	Clean-contaminated	5 (22.7)	17	
	Contaminated	6 (35.3)	11	
	Dirty	2 (50)	2	

Comorbid condition	Yes	5 (41.7)	7	39.765, 0.000
	No	11 (19.6)	45	

Postoperative hospital stay	≤5 days	4 (10.8%)	33	0.529, 0.462
	>5 days	12 (38.7%)	19	

Timing of prophylaxis administration	Early	1 (14.3)	6	21.746, 0.000
	Pre	4 (15.4)	22	
	Peri	0 (0)	5	
	Post	8 (27.6)	21	

Duration of prophylaxis	≤1 day	4 (25)	12	0.54, 0.3021
	>1 day	9 (20.93)	34	

Presence of prophylaxis	Yes	13 (22.03)	46	36.765, 0.00
	No	3 (33.33)	6	

**Table 6:** The association between selected risk factors and surgical site infections.

Variable		Frequency		OR (95% CI)	*p* value
		Yes	No		
Duration of procedure	≤1 hr	9	34	Reference	0.029
	>1 hr	7	18	2.125 (0.659-6.428)	

Wound classification	Clean	3	22	Reference	0.002
	Clean-contaminated	5	17	1.833 (0.204-16.512)	
	Contaminated	6	11	3.4 (0.377-30.655)	
	Dirty	2	2	7.33 (0.734-73.248)	

Comorbid condition	No	11	45	Reference	0.000
	Yes	5	7	5 (0.796-13.325)	

Timing of prophylaxis administration	Pre	4	22	Reference	0.00
	Early	1	6	3.692 (0.373-36.567)	
	Peri	0	5	3.385 (0.85-13.484)	
	Post	8	21	9.94 (0.95-18.34)	

Prophylaxis	Yes	13	46	Reference	0.00
	No	3	6	2.000 (0.388-8.06)	

## Data Availability

The data used to support the findings of this study are included within the article.

## Abbreviations

CDC: Center for Disease Control and Prevention
DRH: Dessie Referral Hospital
SAP: Surgical antibiotic prophylaxis
SSI: Surgical site infection
OR: Odds ratio
IV: Intravenous
PO: Oral
SD: Standard deviation.

## References

[1] Lizioli A., Privitera G., Alliata E. (2003). Prevalence of nosocomial infections in Italy: result from the Lombardy survey in 2000. *Journal of Hospital Infection*.

[2] Malone D. L., Genuit T., Tracy J. K., Gannon C., Napolitano L. M. (2002). Surgical site infections: reanalysis of risk factors. *Journal of Surgical Research*.

[3] Culver D. H., Horan T. C., Gaynes R. P. (1991). Surgical wound infection rates by wound class, operative procedure, and patient risk index. *The American Journal of Medicine*.

[4] Gedebou M., Habte-Gabr E., Kronvall G., Yoseph S. (1988). Hospital-acquired infections among obstetric and gynaecological patients at Tikur Anbessa Hospital, Addis Ababa. *Journal of Hospital Infection*.

[5] Brown S., Kurtsikashvili G., Alonso-Echanove J. (2007). Prevalence and predictors of surgical site infection in Tbilisi, Republic of Georgia. *Journal of Hospital Infection*.

[6] Abula T., Kedir M. (2004). The pattern of antibiotic usage in surgical in-patients of a teaching hospital, northwest Ethiopia. *Ethiopian Journal of Health Development*.

[7] Munckhof W. (2005). Antibiotics for surgical prophylaxis. *Australian Prescriber*.

[8] Mawalla B., Mshana S. E., Chalya P. L., Imirzalioglu C., Mahalu W. (2011). Predictors of surgical site infections among patients undergoing major surgery at Bugando Medical Centre in Northwestern Tanzania. *BMC Surgery*.

[9] Belda F. J., Aguilera L., García de la Asunción J. (2005). Supplemental perioperative oxygen and the risk of surgical wound infection: a randomized controlled trial. *Journal of the American Medical Association*.

[10] McGowan J. E. (1991). Cost and benefit of perioperative antimicrobial prophylaxis: methods for economic analysis. *Clinical Infectious Diseases*.

[11] Misra A. K., Gupta R., Bedi J. S., Narang M., Garg S., Mail I. (2015). Antibiotic prophylaxis for surgical site infection: need of time. *Health*.

[12] Gessler M., Nkunya M. H., Mwasumbi L. B., Heinrich M., Tanner M. (1994). Screening Tanzanian medicinal plants for antimalarial activity. *Acta Tropica*.

[13] Ussiri E., Mkony C., Aziz M. (2004). Sutured and open clean-contaminated and contaminated laparotomy wounds at Muhimbili National Hospital: a comparison of complications. *East and Central African Journal of Surgery*.

[14] Classen D. C., Evans R. S., Pestotnik S. L., Horn S. D., Menlove R. L., Burke J. P. (1992). The timing of prophylactic administration of antibiotics and the risk of surgical-wound infection. *New England Journal of Medicine*.

[15] Apanga S., Adda J., Issahaku M., Amofa J., Mawufemor K. R. A., Bugri S. (2014). Post-operative surgical site infection in a surgical ward of a tertiary care hospital in Northern Ghana. *International Journal of Research in Health Sciences*.

[16] Singh M. P., Brahmchari S., Banerjee M. (2013). Surgical site infection among postoperative patients of tertiary care centre in Central India-a prospective study. *Asian Journal of Biomedical and Pharmaceutical Sciences*.

[17] Tekie K. (2008). *Surgical wound infection in Tikur Anbessa hospital with special emphasis on Pseudomonas aeruginosa. Unpublished MSc thesis in medical microbiology*.

[18] Nobandegani Zinat M., Doulatabad Shahla N., Masoumeh R., Ardeshir A. (2011). Surgical site infection incidence after a clean-contaminated surgery in Yasuj Shahid Beheshti Hospital, Iran. *Investigación y Educación en Enfermería*.

[19] Hernandez K., Ramos E., Seas C., Henostroza G., Gotuzzo E. (2005). Incidence of and risk factors for surgical-site infections in a Peruvian Hospital. *Infection Control & Hospital Epidemiology*.

[20] Gottrup F., Melling A., Hollander D. A. (2005). An overview of surgical site infections: aetiology, incidence and risk factors. *EWMA Journal*.

[21] Habte-Gabr E., Gedebou M., Kronvall G. (1988). Hospital-acquired infections among surgical patients in Tikur Anbessa Hospital, Addis Ababa, Ethiopia. *American Journal of Infection Control*.

[22] Amoran O. E., Sogebi A. O., Fatugase O. M. (2014). Rates and risk factors associated with surgical site infections in a tertiary care center in South-Western Nigeria. *International Journal of Tropical Disease & Health*.

[23] Network SIG (2000). *Antibiotic Prophylaxis in Surgery*.

[24] Dellinger E. P., Gross P. A., Barrett T. L. (1994). Quality standard for antimicrobial prophylaxis in surgical Procedures. *Infection Control and Hospital Epidemiology*.

[25] Akoko L., Mwanga A., Fredrick F., Mbembati N. (2012). Risk factors of surgical site infection at Muhimbili National Hospital, Dar es Salaam, Tanzania. *East and Central African Journal of Surgery*.

[26] Miliani K., L'Hériteau F., Astagneau P., INCISO Network Study Group (2009). Non-compliance with recommendations for the practice of antibiotic prophylaxis and risk of surgical site infection: results of a multilevel analysis from the INCISO Surveillance Network. *Journal of Antimicrobial Chemotherapy*.

[27] Hawn M. T., Itani K. M., Gray S. H., Vick C. C., Henderson W., Houston T. K. (2008). Association of Timely Administration of Prophylactic Antibiotics for Major Surgical Procedures and Surgical Site Infection. *Journal of the American College of Surgeons*.

